# The British Journals, &c.: Midwifery, and Diseases of Women and Children

**Published:** 1842-07

**Authors:** 


### MIDWIFERY, AND DISEASES OF WOMEN AND CHILDREN.

CASE OF TWINS WITH UNION OF THE BODIES OF THE CHILDREN. This was the case of a woman, about thirty years of age, and who was pregnant for the second time. On examination, after the labour had made considerable progress, one head was found protruded, and in the recess, between the chin and breast, a second was in progress of expulsion, which last was pressed so firmly on the throat of the former, as to produce great congestion, the colour of the skin changing from a reddish hue to a deep purple, and the tongue being forced beyond the lips. *Both children were expelled together;* no cry was uttered by either, nor was any movement made by them; nor could pulsation at the precordial region be detected. The mother made a good recovery. The children, both males, were united by the chest anteriorly, the union extending a few inches towards the side. The abdominal parietes of both children were deficient below the umbilicus; the viscera, having consequently no support, hung down in front. There was only one source of nourishment from the parent, and one liver, which was larger than usual. There were two hearts, *inclosed in one pericardium;* that supplying the child whose head was born first, was the smaller; the other about the normal size. Each had the usual number of hands and feet, and the organs of generation in both were perfect.—Dr. SKIPTON, *Dublin Medical Press,* No. 177. *May* 25, 1842.

Birth of a Living Child, on the 179th day. The mother of this child was married on the 22d of July, 1839 ; had menstruated duly the week before; and was well at the time of her marriage. Her menses did not return after that event. Premature labour came on, on the 17th of January, 1840, and on the 18th, she was delivered of a female child. There were no nails on the fingers or toes of the infant; a thick dark down covered the head instead of hair; the skin was unusually florid and thin, the extremities imperfectly developed, the membrana pupularia were entire. Before it sucked it was shrivelled and covered with down similar to that on its head. It was too feeble to grasp the mother's nipple, and was fed for the first three weeks by milk taken from the breast, and introduced into its mouth by a quill or spoon. It was not weighed or measured until forty days after its birth. It was then nearly the weight of three pounds; and the length of thirteen inches. The centre of the body was about an inch above the umbilicus. It died on the 29th of May, after two days' illness of measles. Before that period, the nails had grown, and the down had almost disappeared from the body.

William Tait, Esq., *Lancet*, No. iv. *April* 23, 1842.

---

Breech Case. Dr. Reid on being called to a woman in her eighth labour, found a midwife had been attempting to extract the child, by pulling at the body, which was already born; the head being still retained. No endeavour had been made to adjust the head. Dr. Reid, on making examination, found the face in the hollow of the sacrum, and on introducing the forefinger into the mouth, and gently depressing the chin, the head was expelled immediately without the smallest difficulty. Great ignorance had been displayed in this case, and neglect of the rule "arte non vi," for on examining the body of the child, it was found not only extensively bruised, but to have both its thigh bones fractured. Dr. Reid has had, from June 1840, to February 1842, 670 cases, producing 678 children, 351 males, 327 females, of which 71 were still-born; 44 premature births; 11 in a decomposed state.—Dr. James Reid, *Medical Gazette*, No. xx. *March* 11, 1842.

---

An Enquiry into the Results of Puncture of the Head in Cases of Chronic Hydrocephalus. This is a most elaborate paper, highly creditable to its learned author, and claiming the perusal of all likely to be called on to treat the disease which is the subject of it. It contains a tabular analysis of fifty-six cases of hydrocephalus in which puncture was performed, giving in each case the sex, age, symptoms before puncture, size of the head, number and date of the punctures, quantity of the fluid evacuated, subsequent progress of the case, date of the report and lastly the authority. The following extracts give a brief summary of the principal results of the enquiry and the conclusions deduced by Dr. West.

The writer has found mention of 63 cases of chronic hydrocephalus in which the cranium was punctured. In two of these cases, however, the puncture was accidental, while in five instances the results were not such as would justify classing the cases either as fortunate or unsuccessful. Fifty-six cases then remain, in 40 of which the patients died, while in 16 they are alleged to have recovered; or, in other words, the proportion of recoveries to deaths was as 1 : 2·5, and as 1 : 3·5 of the total cases. These results, though considerably less favorable than those obtained by Dr. Conquest, still appear at first sight to afford ample justification of the operation; but the particulars contained in the table will, perhaps, in some degree modify such an opinion.

It would have been interesting to have been made acquainted with the circumstances to which the brilliant success of the operation in Dr. Conquest's hands is attributable. But unfortunately, no data are given in 15 out of 19 cases, beyond the mere statement of the number of punctures, and the quantity of fluid removed. The age of the patient, the duration of the disease, the symptoms attending it, the size of the head, and the condition of the intellectual faculties before and after the operation, are not noticed. We are left in perfect ignorance as to the time which elapsed before each patient was reported as cured; and yet, on grounds so slender, an impression has got abroad in this country and elswhere that paracentesis capitis is a means to which recourse may be had in cases of chronic hydrocephalus, with a well-founded expectation of success.

In 30 of the 40 cases, (in which the operation was unsuccessful,) the interval which elapsed between the performance of the operation and the patient's death is stated; and it appears that the deaths after the first puncture were as follows;

| Deaths. | | | Average duration of life after the puncture. |
|---|---|---|---|
| 6 | within | 4 days | 53 hours. |
| 6 | " | 14 days | 6 days 8 hours. |
| 3 | " | 1 month | 20 days 16 hours. |
| 9 | " | 3 months | 56 days 10 hours. |

Of the remaining 6, only 1 survived the puncture 6 months; and the average duration of life in each of these was 3 months 4 days 12 hours. In 18 of these patients, the operation was performed more than once; but in no instance did the children survive the last puncture more than 35 days, while the average duration of life was 12 days 22 hours.

The instances, then, in which life was prolonged by the operation appear to be very few, and the cases in which any reasonable prospect of the patient's recovery existed after a week had elapsed from the first performance of the puncture, are still fewer. The table shows that sometimes the puncture was followed by an almost immediate aggravation of the cerebral symptoms, and by death. Usually, however, a degree of apparent improvement followed the puncture, but the fluid soon collected again, and less marked relief followed the second operation. With its repetition the quantity of fluid increased, and while the size of the head continued undiminished, or even grew larger, the body of the patient became emaciated: and death either took place from exhaustion, or cerebral symptoms came on, and life was terminated by coma or convulsions.

If the symptoms observed during life yield little encouragement to resort to the operation, the appearances disclosed after death afford a powerful argument against it. An account is given of the post-mortem examination of 26 cases. In every instance fluid, sometimes in considerable quantity, was contained within the ventricles or in the cavity of the cranium, and the substance of the brain was softened and attenuated. But, in addition to these appearances, there existed in 16 cases, serious organic disease or malformation of the brain itself, though no symptom during life had betrayed the existence of a condition which mechanical interference could only aggravate.—CHARLES WEST, M.D. *Med. Gaz. April* 15, 1842.

---

ARTIFICIAL PREMATURE LABOUR. A woman, married seven years, and aged twenty-eight, has been pregnant six times. Having a slightly contracted pelvis and generally large children, she has never been able to give birth to a fœtus at the full period, without embryotomy. Mr. Braine desired her to inform him of the fact of her pregnancy at the end of the 7th month, on the next occasion of her being in that condition, which she did. Mr. Braine then waited until she was somewhat advanced in the 8th month, when he punctured the membranes, thirty-two hours after which operation, a living child was born. Both mother and child did well.—Mr. BRAINE, *Prov. Med. and Surg. Jour.* No. iii. *April* 23, 1842.

NEW MODE OF ACCELERATING LABOUR. The practice consists simply, in imitating the influence of the child's head, or membranes on the natural passages, and thus producing expulsive efforts by reflex sympathy. This is accomplished by introducing the finger or fingers as far as the point of the os coccygis, and passing them downwards along the whole surface of the vagina, so as to give the sensation of distension.—Mr. STANILAND, *Provincial Med. and Sur. Journal,* No. xxvi. *March* 26, 1842.

